# Synthesis and characterization of the ‘Japanese rice-ball’-shaped Molybdenum Blue Na_4_[Mo_2_O_2_(OH)_4_(C_6_H_4_NO_2_)_2_]_2_[Mo_120_Ce_6_O_366_H_12_(OH)_2_(H_2_O)_76_]∼200H_2_O

**DOI:** 10.1107/S2053229622003369

**Published:** 2022-04-20

**Authors:** Emir Al-Sayed, Elias Tanuhadi, Gerald Giester, Annette Rompel

**Affiliations:** a Universität Wien, Fakultät für Chemie, Institut für Biophysikalische Chemie, Althanstrasse 14, 1090 Wien, Austria; b Universität Wien, Fakultät für Geowissenschaften, Geographie und Astronomie, Institut für Mineralogie und Kristallographie, Althanstrasse 14, 1090 Wien, Austria

**Keywords:** polyoxomolybdate, cerium, hybrid organic–inorganic, nanocluster, crystal structure, Molybdenum Blue

## Abstract

The hybridized lanthanide-containing molybdenum blue (**Ln-MB**) wheel Na_4_[Mo_2_O_2_(OH)_4_(C_6_H_4_NO_2_)_2_]_2_[Mo_120_Ce_6_O_366_H_12_(OH)_2_(H_2_O)_76_]∼200H_2_O ({Mo_2_(C_6_H_4_NO_2_)_2_}_2_{Mo_120_Ce_6_}) was assembled in an aqueous one-pot synthesis. The **Ln-MB** was hybridized with 2-picolinic acid through the generation of the organometallic counter-ion [Mo_2_O_2_(OH)_4_(C_6_H_4_NO_2_)_2_]^2+^. Control experiments demonstrated that the position of the carb­oxy­lic acid group (2-position to the N atom) in the hybridization com­ponent is critical in yielding single crystals of **Ln-MB**. In addition to single-crystal X-ray diffraction (XRD) analysis, which revealed a ‘Japanese rice-ball’-shaped **Ln-MB** as the anion, elemental analyses, IR spectroscopy, and thermogravimetric analysis (TGA) were performed to confirm its structure and com­position. Bond-valence-sum calculations (BVS) revealed that {Mo_2_(C_6_H_4_NO_2_)_2_}_2_{Mo_120_Ce_6_} is com­posed of a 24-electron re­duced anionic ring, which was confirmed by Vis–NIR spectroscopy.

## Abstract 2

The synthesis and crystal structure of a ‘Japanese rice-ball’-shaped Molybdenum Blue hybridized organically with 2-picolinic acid is presented. In addition to single-crystal X-ray analysis, the title com­pound was characterized with IR spectroscopy and elemental analyses to reinforce its framework structure, thermogravimetric analysis to qu­antify the water content, and Vis–NIR spectroscopy to determine the degree of reduction of the nanocluster.

## Introduction

Polyoxometalates (POMs) are polynuclear oxo-bridged metal oxide clusters primarily com­posed of early transition metals in their highest oxidation states (Pope, 1987 ▸; Gumerova & Rompel, 2020 ▸). The early transition-metal ions (*M^n+^*) are commonly Mo^V/VI^, W^V/VI^, V^IV/V^, Nb^V^, or Ta^V^, which form {*M*O_*x*_} (*x* = 4–7) polyhedra that are typically linked together *via* corner- and edge-shared O atoms (Pope, 1987 ▸). Their structural diversity (size, shape, and com­position) and unique properties give rise to a plethora of potential applications from medicine (Stephan *et al.*, 2013 ▸; Yamase, 2005 ▸; Bijelic *et al.*, 2019 ▸; Tanuhadi *et al.*, 2020 ▸) to catalysis (Al-Sayed *et al.*, 2021 ▸; Chen *et al.*, 2021 ▸) and macromolecular crystallography (Bijelic *et al.*, 2015 ▸; Mauracher *et al.*, 2014*a* ▸,*b* ▸; Breibeck *et al.*, 2019 ▸).

Molybdenum Blues (**MB**s) are giant POMs with the general formula [*X_a_Y_b_* H_*c*_Mo^VI^_*x*_Mo^V^_*y*_O_*z*_(H_2_O)_*v*_]^*n*−^ (*a* = number of organic ligands; *b* = number of metallic heteroelements; *c* = degree of protonation; *x* and *y* = number of unreduced and reduced molybdenum, respectively; *z* = number of O atoms; *v* = number of coordinated water; *n* = resulting charge of the nanosized scaffold) (Al-Sayed & Rompel, 2022 ▸), with versatile topologies and high structural flexibility. **MB**s are commonly con­structed by generating and combining the virtual building blocks {MoO_6_} ({Mo_1_}) and {Mo_2_O_11_} ({Mo_2_}), and the funda­mental building block {Mo_8_O_35_} ({Mo_8_}) with the pen­ta­gonal unit {MoO_7_Mo_5_O_20_} ({Mo(Mo)_5_}) (Müller & Gouzerh, 2012 ▸). {Mo_1_}, {Mo_2_}, and {Mo_8_} are formed upon acidification (pH ≤ 4.5) (Shishido & Ozeki, 2008 ▸) of an orthomolybdate ([MoO_4_]^2−^) solution (∼20–30 m*M* for lan­tha­nide-containing MBs in a one-pot synthesis approach) and subsequent addition of a reducing agent (*e.g.* N_2_H_4_ with *c* ∼ 1 m*M*) (Müller & Roy, 2002 ▸).

The coordinative attachment of organic ligands onto {Mo_2_} building blocks, the {Mo_2_} substitution by metal ions of suitable size, such as lanthanide ions (**Ln^III^**), and the incorporation of long-chain organic surfactants as charge-balancing cations enable structural modifications and changes in the physical properties (*e.g.* solubility) of the MB cluster. The modifications include organically functionalized nanocavities, which allow the stabilization of anionic templates in their centre *via* hydrogen bonds, as well as the construction of mol­ecular shapes (‘Japanese rice-ball’, ‘egg’ and ellipsoid) that deviate from the {Mo_154–*x*_} (*x* = number of defect sites) wheels (circle-shaped) (Al-Sayed & Rompel, 2022 ▸). The introduction of long-chain organic surfactants (*e.g.*, dido­decyl­dimethyl­am­monium, DDMA) increases the hydro­phobicity and allows the polarity of the cluster to be modulated (Polarz *et al.*, 2001 ▸; Jing *et al.*, 2013 ▸). The **Ln-MB** crystal structure library is rather small, with about 30 crystal structures of **Ln-MB** ring systems (of which five are ‘Japanese rice-ball’-shaped **Ln-MB**s; Table 1 ▸) reported to date (as of January 2022).

Previously, tryptophan has been utilized as a hybridizing ligand for functionalizing the inner ring of an ellipsoidal **Ln-MB**, yielding the cluster {Mo_124_Ce_4_(tryptophan)_4_}, featuring unprecedented kynurenine counter-cations as a result of tryptophan oxidation (Xuan *et al.*, 2018 ▸) that occurred *in situ* during the self-assembly of {Mo_124_Ce_4_(tryptophan)_4_}. Fol­lowing the kynurenine pathway (Tan *et al.*, 2012 ▸), which is a metabolic pathway and starts with the oxidation of tryptophan, the catabolite 2-picolinic acid was identified as a bidentate chelating agent. Herein 2-picolinic acid is utilized as an {Mo_2_}-hybridizing ligand, yielding the isolation of the new **Ln-MB** Na_4_[Mo_2_O_2_(OH)_4_(C_6_H_4_NO_2_)_2_]_2_[Mo_120_Ce_6_O_366_H_12_(OH)_2_(H_2_O)_76_]∼200H_2_O featuring the organometallic coun­ter-cation [Mo_2_O_2_(OH)_4_(C_6_H_4_NO_2_)_2_]^2+^.

## Experimental

### Synthesis and crystallization

25 ml of a 3.6 m*M* Ce^III^ stock solution [0.9 mmol CeCl_3_·7H_2_O (0.335 g) dissolved in 250 ml H_2_O] were com­bined with 25 ml of a 40 m*M* [MoO_4_]^2−^ stock solution [10 mmol Na_2_MoO_4_·2H_2_O (2.42 g) dissolved in 250 ml H_2_O]. Following the addition of 2-picolinic acid (0.14 mmol, 0.0172 g), the solution was reduced with 0.5 ml of an aqueous hydrazine ([N_2_H_4_]·2HCl) solution (0.1 *M*), acidified with 4.5 ml HClO_4_ (1 *M*) to pH ∼1.4, and subsequently heated between 85–90 °C for 1.5 h in an Erlenmeyer flask covered with a watch glass. The resulting clear deep-blue solution was left to crystallize in the open Erlenmeyer flask for two weeks at room temperature. Deep-blue block-shaped crystals were filtered off, washed with ice-cold H_2_O and air-dried (yield: 45 mg, 22.5%, based on Mo). Elemental analysis calculated (%): C 1.18, H 2.44, N 0.23, Na 0.38, Mo 48.77, Ce 3.45; found: C 1.37, H 1.45, N 0.39, Na 0.35, Mo 51.5, Ce 4.0. FT–IR (cm^−1^): 3252 (*br*), 1606 (*m*), 1411 (*m*), 1092 (*m*), 967 (*m*), 904 (*m*), 866 (*m*), 806 (*s*), 746 (*s*), 620 (*s*), 528 (*s*).

### Refinement

Crystal data, data collection and structure refinement details are summarized in Table 2 ▸. C—H bond lengths were constrained to 0.95 Å for pyridine-2-carboxyl­ate C—H groups and refined in riding modes, with *U*_iso_(H) values set to 1.2*U*_eq_(C). SADI (equal distance) restraints were applied to the C—N and C—C bonds of one pyridine-2-carboxyl­ate ring (N2—C7, C8—C7, C9—C8, C10—C9, C10—C11 and N2—C11) and to the C—O bonds C12—O224 and C6—O225. The bond between the aromatic carbon C1 and the carboxyl­ate carbon C6 (C1—C6) was restrained to 1.43 (2) Å. In addition, all C atoms and some O atoms of the nanoring had to be refined using the constraint of equivalent anisotropic displacement parameters (EADP). One of two sodium ions in the asymmetric unit was refined with two positions (Na2 and Na3), each with 0.5 occupation factor. The refining of (disordered) H_2_O mol­ecules with positioned H atoms proved unachievable due to the high number of (disordered) H_2_O mol­ecules. Residual electron density arising from disordered H_2_O mol­ecules could be identified during crystal structure refinement. Considering the disorder of the H_2_O mol­ecules preventing a satisfactory refinement the corresponding electron densities were described employing a solvent mask to stabilize the refinement, and the qu­antity of H_2_O mol­ecules determined with TGA was entered into the CIF file.

### Elemental analyses

The content of C/H/N/O was determined using an EA 1108 CHNS-O elemental analyzer from Carlo Erba Instruments at the Mikroanalytisches Laboratorium, Faculty of Chemistry, University of Vienna. The determination of Na/Mo/Ce was performed by Technische Universität Hamburg, Zentrallabor Chemische Analytik, Hamburg, Germany.

### Vis–NIR spectroscopy

Vis–NIR spectroscopy was carried out at 298 K on a Shimadzu UV-2401PC spectrophotometer using a quartz cuvette with a 1.0 cm optical path length.

### Thermogravimetric analysis (TGA)

TGA was conducted using a thermal analyzer (TA instruments model Q50, USA). The sample, having an initial mass of ∼15 mg, was subjected to a temperature range of 298–1173 K at a heating rate of 5 K min^−1^.

### Attenuated total reflectance Fourier-transform infrared spectroscopy (ATR FT–IR)

All FT–IR spectra were recorded on a Bruker Vertex 70 IR spectrometer equipped with a single-reflection diamond ATR unit. Frequencies are given in cm^−1^ and intensities are denoted as *w* = weak, *m* = medium, *s* = strong, and *br* = broad.

## Results and discussion

**Figure 1:**
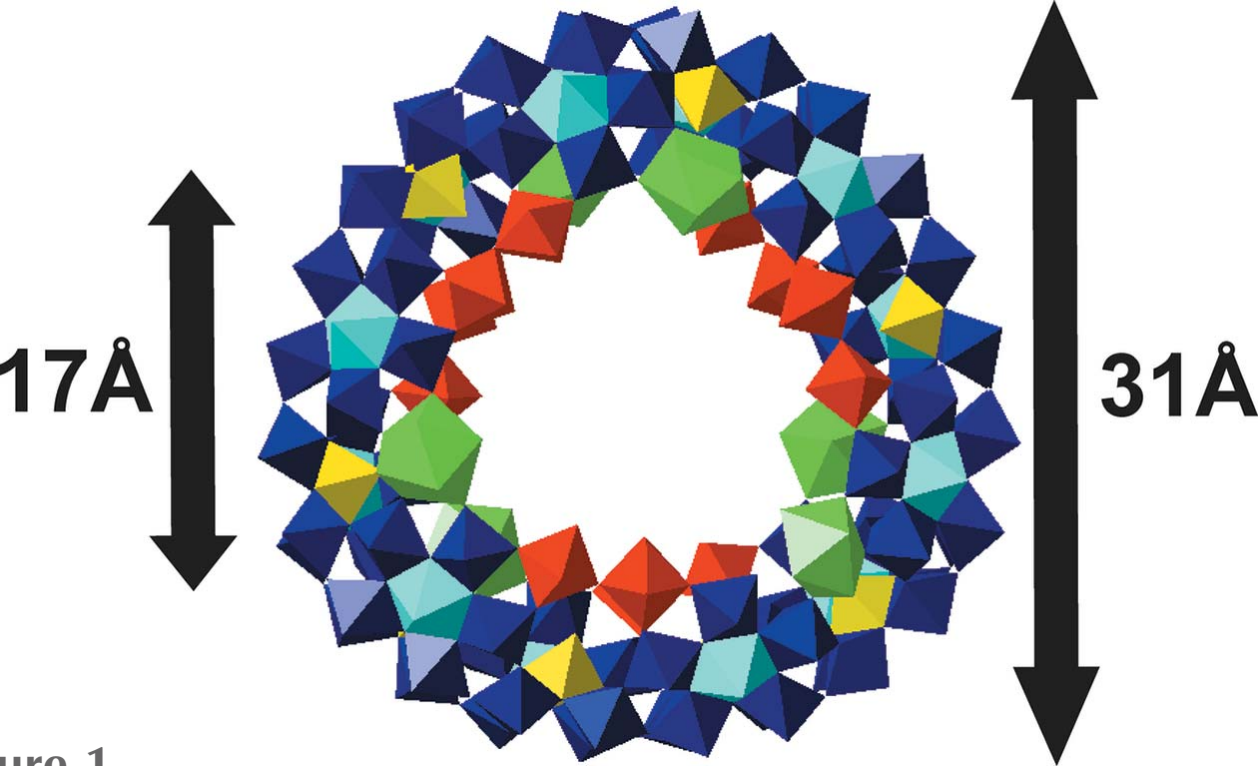
Polyhedral representation [and inner (left) and outer (right) diameters of the respective ring/rim] of the ‘Japanese rice-ball’ in {Mo_2_(C_5_H_5_N)_2_}_2_{Mo_120_Ce_6_}. Colour code: {MoO_6_} yellow, {Mo_2_O_11_} red, {Mo_8_O_35_} blue, with the central {MoO_7_} unit in cyan, and {CeO_9_} green.

**Figure 2:**
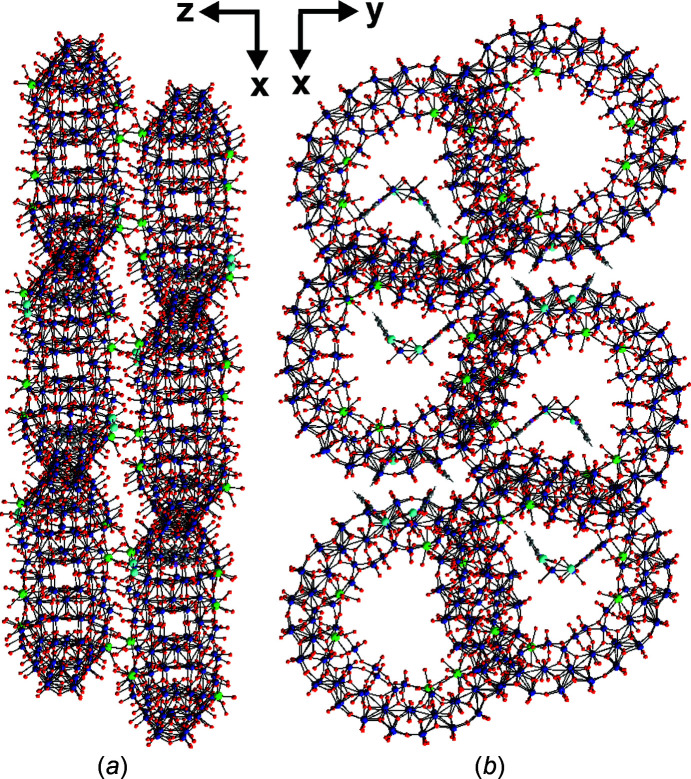
Ball-and-stick representation of the packing mode of {Mo_2_(C_6_H_4_NO_2_)_2_}_2_{Mo_120_Ce_6_} along the (*a*) *y* axis and (*b*) *z* axis. Colour code: Mo blue, Ce green, O red, Na turquoise, C grey, and N pink.

**Figure 3:**
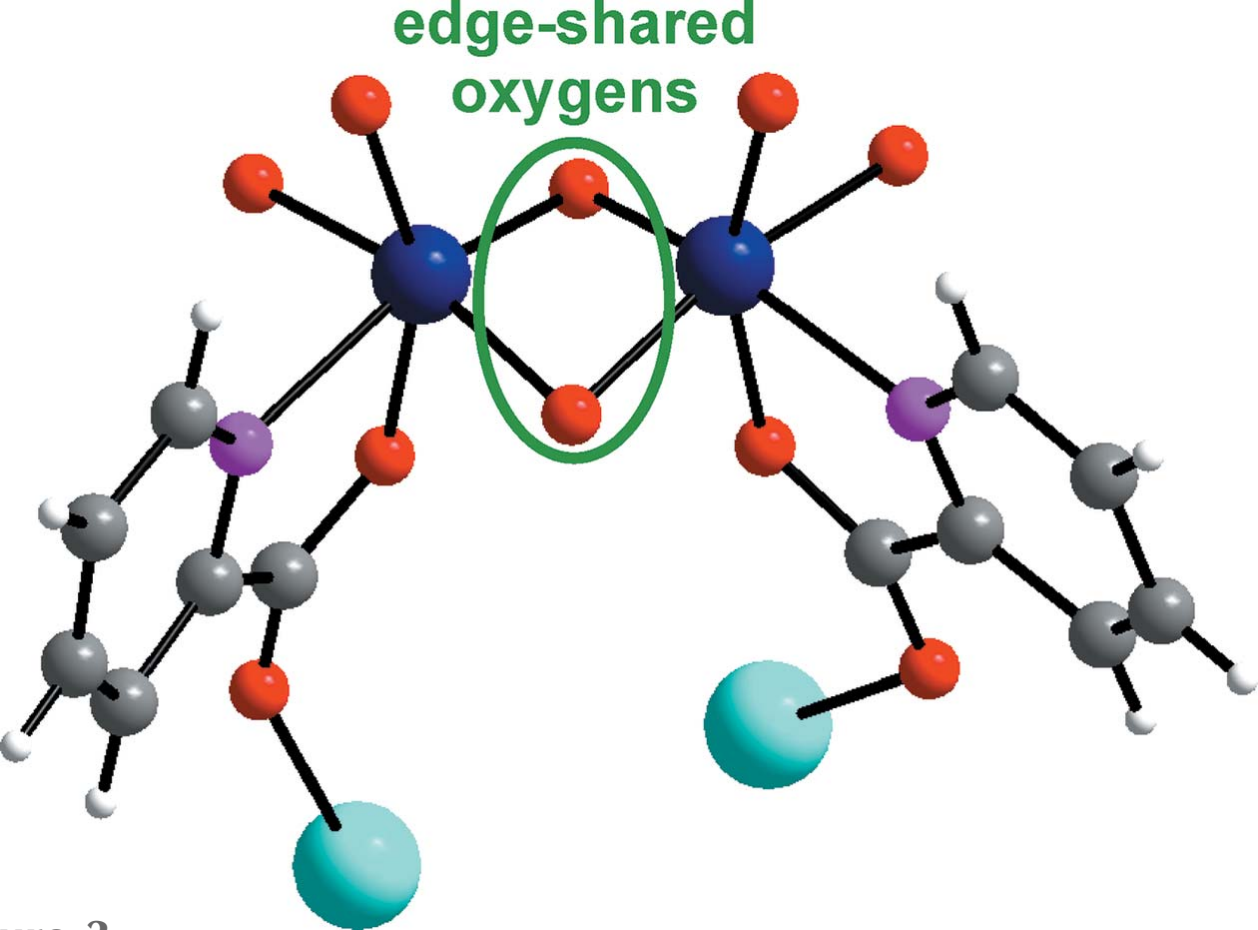
Ball-and-stick representation of the charge-balancing cation [Mo_2_O_2_(OH)_4_(C_6_H_4_NO_2_)_2_]^2+^ ({Mo_2_(C_6_H_4_NO_2_)_2_}^2+^) coordinated to Na^+^ ions. Colour code: Mo dark blue, C grey, N pink, O red, H white, and Na turquoise.

The 2-picolinic acid/Ce/Mo ratio (0.14/0.9/1) was critical for producing single crystals of Na_4_[Mo_2_O_2_(OH)_4_(C_6_H_4_NO_2_)_2_]_2_[Mo_120_Ce_6_O_366_H_12_(OH)_2_(H_2_O)_76_]∼200H_2_O ({Mo_2_(C_6_H_4_NO_2_)_2_}_2_{Mo_120_Ce_6_}). When the concentration of 2-picolinic acid was lower (0.03–0.12 mol equivalents), either small weakly scattering crystals formed or the reaction solution remained clear with no crystals forming. {Mo_2_(C_6_H_4_NO_2_)_2_}_2_{Mo_120_Ce_6_} is a ‘Japanese rice-ball’-shaped com­plete **Ln-MB** ring system, which crystallizes in the space group *C*2/*c*. The inner and outer diameters of the **Ln-MB** anion {Mo_120_Ce_6_} are ∼17 and ∼31 Å, respectively (Fig. 1 ▸). The {Mo_124_Ce_6_} scaffold is com­posed of 12 {Mo_1_}, 6 {Mo_2_}, 12 {Mo_8_}, and 6 {Ce} (= {Ce^III^O_9_}) building units. In the ‘Japanese rice-ball’ {Mo_2_(C_6_H_4_NO_2_)_2_}_2_{Mo_120_Ce_6_}, six {Mo_2_} groups are replaced by six {Ce^III^} groups. The average size of the incorporated {Ce^III^} in the inner ring (O—Ce^III^—O) is 4.8 Å, while the corner-sharing {Mo_2_} units (O—Mo—O—Mo—O) are 7.3 Å, forcing the cluster into a ‘more contracted’ architecture exhibiting an irregular ring shape and a lower symmetry (*D*_3_) com­pared to that of the ideal circular parent structure {Mo_154_} (*D*_7*d*_) (Müller *et al.*, 1996 ▸). All cerium ions on both the upper and lower surfaces of {Mo_120_Ce_6_} are trivalent and exhibit tricapped trigonal prismatic coordination spheres (Fig. 1 ▸). Each Ce^III^ ion is coordinated by five water mol­ecules ({Ce^III^(H_2_O)_5_}) and is linked to the **Ln-MB** scaffold *via* six μ_2_-O atoms. The negative charge of 8− of the ‘Japanese rice-ball’ is balanced by two [Mo_2_O_2_(OH)_4_(C_6_H_4_NO_2_)_2_]^2+^ ({Mo_2_(C_6_H_4_NO_2_)_2_}^2+^) organometallic cations and four Na^+^ ions (Fig. 2 ▸), which are located in the outer shell of the cluster. In {Mo_2_(C_6_H_4_NO_2_)_2_}^2+^, two 2-picolinic acid mol­ecules are coordinated equatorially and axially onto both Mo^VI^ ions, which are linked together *via* monoprotonated edge-shared O atoms (Fig. 3 ▸). Control experiments revealed that neither nicotinic acid nor isonicotinic acid (both isomers of 2-picolinic acid) can yield hybridized **Ln-MB** frameworks under otherwise identical synthetic conditions.

The sum formula of {Mo_2_(C_6_H_4_NO_2_)_2_}_2_{Mo_120_Ce_6_} was determined based on single-crystal X-ray diffraction (XRD), elemental, bond-valence-sum (BVS; Brown, 1981 ▸), and thermogravimetric (TGA) analysis. Furthermore, BVS was carried out to calculate the number of Mo^V^ centres within the **Ln-MB** and UV–Vis–NIR spectroscopy was performed to determine the contribution of each Mo^V^ centre to the overall reduction state of the nanocluster. Due to the low water solubility of {Mo_2_(C_6_H_4_NO_2_)_2_}_2_{Mo_120_Ce_6_}, which is a frequently encountered problem in the case of hybridized **Ln-MB**s (Xuan *et al.*, 2019 ▸; She *et al.*, 2021 ▸), redox titration to determine the number of reduced electrons in {Mo_2_(C_6_H_4_NO_2_)_2_}_2_{Mo_120_Ce_6_} was not feasible.

**Figure 4:**
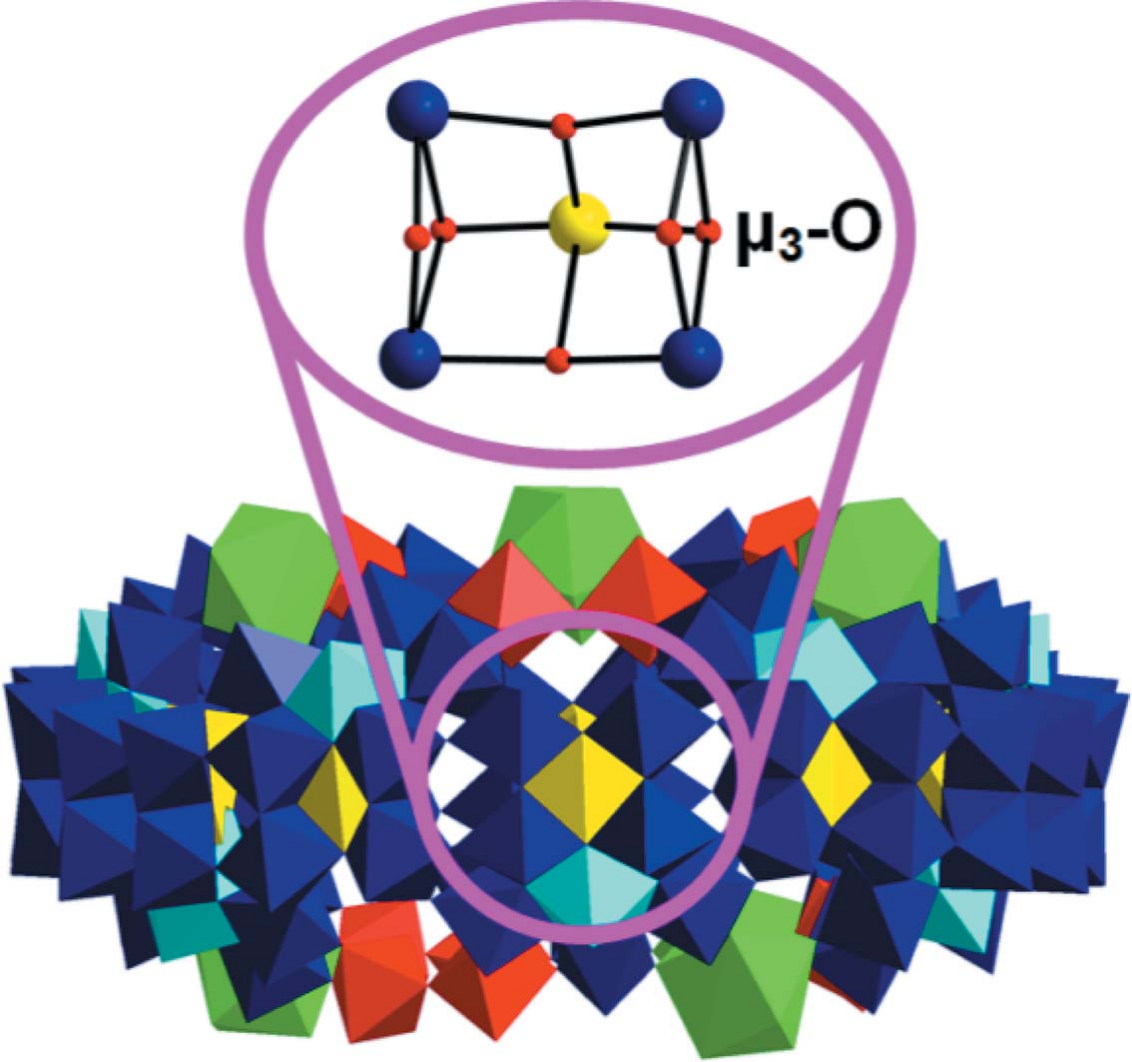
Polyhedral representation of {Mo_2_(C_5_H_5_N)_2_}_2_{Mo_120_Ce_6_}, with one of 12 {Mo_5_O_6_}-incom­plete double-cubane-type com­partments highlighted in a ball-and-stick representation. Colour code: {MoO_6_} yellow, {Mo_2_O_11_} red, {Mo_8_O_35_} blue, with the central {MoO_7_} unit in cyan, {CeO_9_} green, Mo blue and yellow spheres, and O red spheres.

BVS calculations revealed that {Mo_2_(C_6_H_4_NO_2_)_2_}_2_{Mo_120_Ce_6_} is constructed of a 24-electron reduced wheel containing 14 mono- and 76 diprotonated O atoms. 12 monoprotonated O atoms are the μ_3_-O of the 12 {Mo_5_O_6_}-incom­plete double-cubane-type com­partments in the equatorial plane of the wheel (Fig. 4 ▸). They exhibit an average BVS value of 1.2 (= monoprotonation), consistent with previous work (Müller & Serain, 2000 ▸; Xuan *et al.*, 2018 ▸). The average BVS value of the Mo centres in the equatorial plane of the wheel spanning the 12 {Mo_5_O_6_} com­partments is 5.6, demonstrating the presence of two 4*d* electrons delocalized in each com­partment, which is in accordance with previous work (Müller & Serain, 2000 ▸; Xuan *et al.*, 2018 ▸).

**Figure 5:**
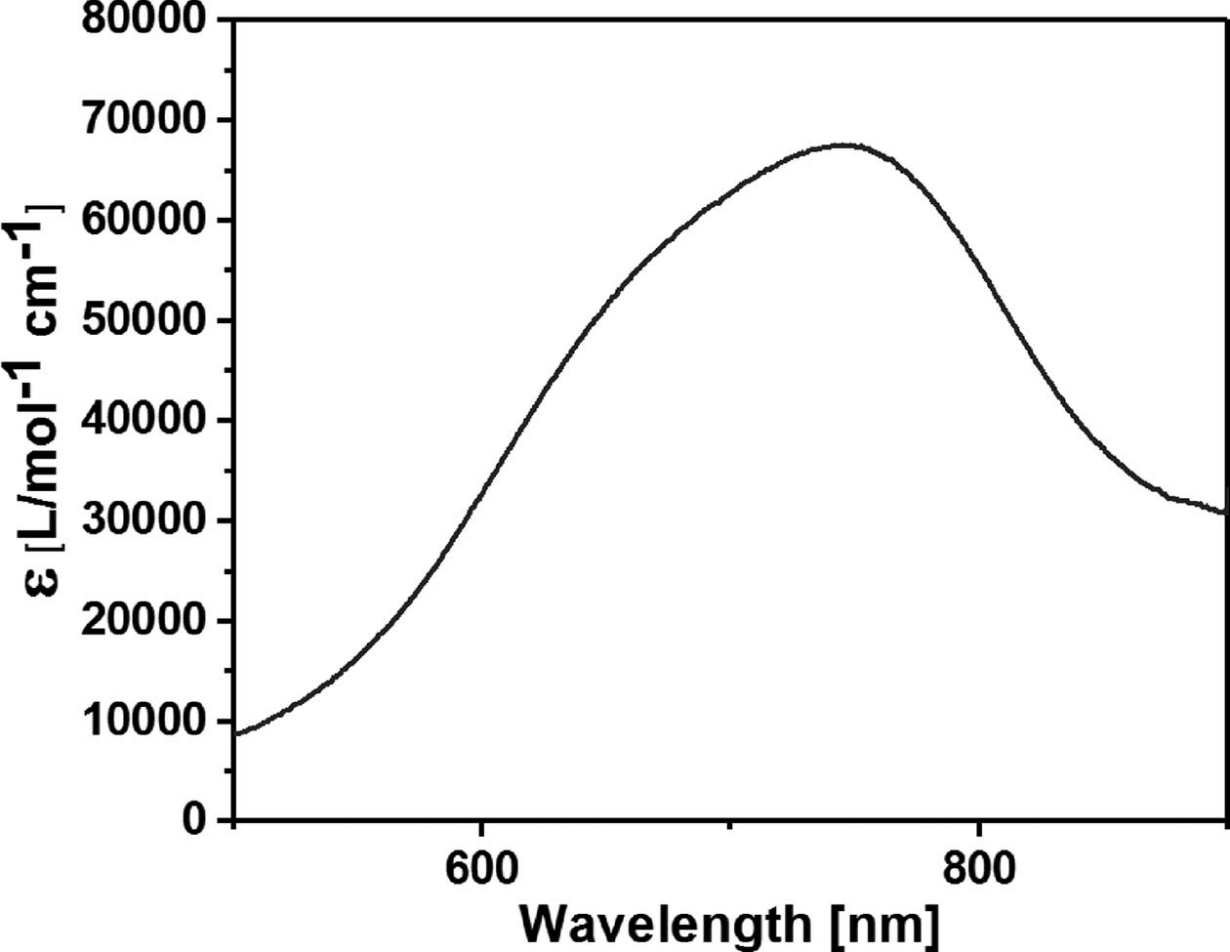
Vis–NIR spectrum of {Mo_2_(C_6_H_4_NO_2_)_2_}_2_{Mo_120_Ce_6_} in 0.5 *M* H_2_SO_4_ (*c* = 1.56 × 10^−5^ mol l^−1^).

The molar extinction coefficient (ɛ_M_) of **Ln-MB**s around 750 nm (in aqueous medium) is associated with the total number of reduced Mo^V^ centres present in the nanocluster (Müller & Gouzerh, 2012 ▸). The ɛ_M_ of {Mo_2_(C_6_H_4_NO_2_)_2_}_2_{Mo_120_Ce_6_} was determined to be 6.76 × 10^4^ l mol^−1^ cm^−1^ (in a 0.5 *M* H_2_SO_4_ solution to ensure com­plete dissolution) which is in the range typical for ‘Japanese rice-ball’-shaped **Ln-MB**s with 24 Mo^V^ centres (2.6–14.4 × 10^4^ l mol^−1^ cm^−1^) (Yamase, 2005 ▸; Duros *et al.*, 2017 ▸). Consequently, the average contribution to the determined ɛ_M_ is approximately 2.82 × 10^3^ l mol^−1^ cm^−1^ per Mo^V^ centre at 745 nm (Fig. 5 ▸), which corresponds to the inter­valence charge transfer between Mo^VI^ and Mo^V^.

**Figure 6:**
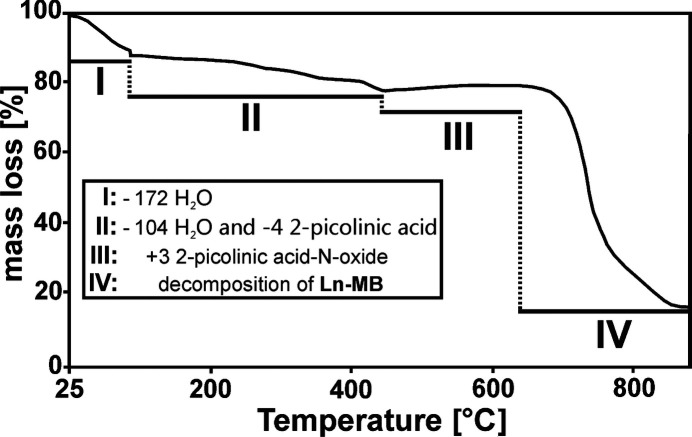
Thermogravimetric curve of {Mo_2_(C_5_H_5_N)_2_}_2_{Mo_120_Ce_6_}, exhibiting three steps (I, II and IV) of weight loss and one step (III) of weight increase.

TGA was carried out to determine the number of crystal, coordinated, and structural water mol­ecules in {Mo_2_(C_6_H_4_NO_2_)_2_}_2_{Mo_120_Ce_6_}. The obtained TG curve shows three main steps (I, II, and IV) of weight losses and one step (III) of weight increase in the range between 25 and 900 °C (Fig. 6 ▸). The first weight loss (∼13%) occurs between 25 and 110 °C, corresponding to ∼172 crystalline H_2_O. The second weight loss (∼10%) takes place between 110 and 473 °C, which can be assigned to ∼104 H_2_O (= 76 coordinated H_2_O + 28 structure H_2_O), and ∼4 2-picolinic acid ligands. The third step (III) emerges between 473 and 630 °C, and represents an increase in weight (∼1.75%) attributed to the oxidation of ∼3 2-pico­linic acid ligands to 2-picolinic acid *N*-oxide by {Mo_2_(C_6_H_4_NO_2_)_2_}_2_{Mo_120_Ce_6_}, which is consistent with previous observations made for Mo^VI/V^-containing POMs of the Keplerate archetype as catalysts for the conversion of picolinic acid derivatives to the corresponding *N*-oxides in excellent yields (Yang *et al.*, 2015 ▸). The fourth and last weight loss (∼75%) occurs between 635 and 900 °C, and is related to the decom­position of the metal oxide framework of **Ln-MB**.

**Figure 7:**
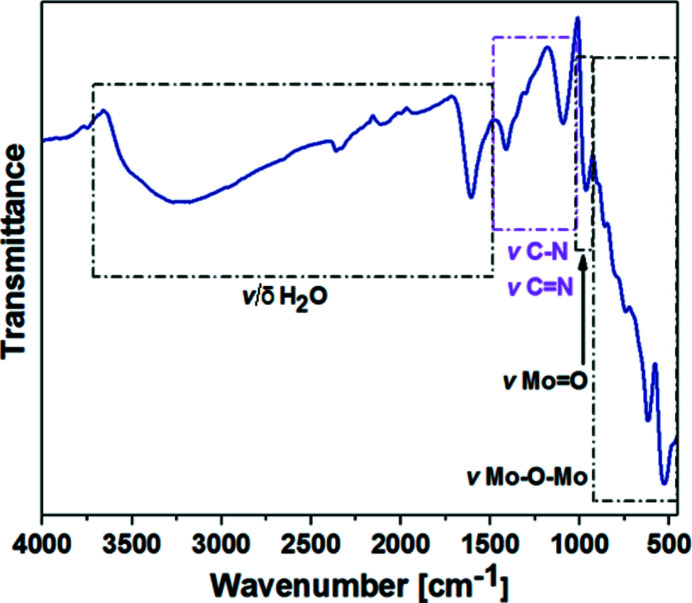
FT–IR spectrum of {Mo_2_(C_6_H_4_NO_2_)_2_}_2_{Mo_120_Ce_6_} in the region between 4000 and 450 cm^−1^.

The FT–IR spectrum of {Mo_2_(C_6_H_4_NO_2_)_2_}_2_{Mo_120_Ce_6_} is depicted in Fig. 7 ▸, with the main vibrational bands listed in Table 3 ▸. The sharp and broad bands in the region between 1606 and 3252 cm^−1^ correspond to the stretching and bending vibrations ν/δ(O—H) of H_2_O. The vibrational bands ν(C—N) and ν(C=N) of 2-picolinic acid emerge between 1092 and 1411 cm^−1^, which are missing in a pure inorganic cerium-containing ‘Japanese rice-ball’ (Duros *et al.*, 2017 ▸). The vib­rational bands ν(C=C) (∼1600 cm^−1^) and ν(=C—H) (∼3000 cm^−1^) of 2-picolinic acid are obscured as they are likely overlaid by the water bands in this particular region. The vibrational band at 967 cm^−1^, which is very sharp and characteristic for molybdenum-based POM structures, is attributed to terminal Mo=O groups. All bands appearing below 967 cm^−1^ correspond to the deformation vibrations ν(Mo—O—Mo) of the Mo—O—Mo bridging units.

## Conclusion

The successful construction of {Mo_2_(C_5_H_5_N)_2_}_2_{Mo_120_Ce_6_} enlarged the sparse crystal structure library of ‘Japanese rice-ball’-shaped **Ln-MB**s. As {Mo_2_}-type building blocks, resulting from the self-assembly process of **Ln-MB** clusters, are organically modifiable, grafting organic ligands onto them yields unique hybridized inorganic–organic **Ln-MB** frameworks. {Mo_2_(C_5_H_5_N)_2_}_2_{Mo_120_Ce_6_} is the first reported ‘Japanese rice-ball’-shaped **Ln-MB** containing a metal–organic charge-balancing unit com­plexed aromatically with 2-picolinic acid in the outer shell.

## Supplementary Material

Crystal structure: contains datablock(s) I, global. DOI: 10.1107/S2053229622003369/ky3215sup1.cif

Structure factors: contains datablock(s) I. DOI: 10.1107/S2053229622003369/ky3215Isup2.hkl

CCDC reference: 2161749

## Figures and Tables

**Table 1:** List of purely inorganic and hybridized inorganic–organic lanthanide-containing Molybdenum Blue wheels exhibiting a ‘Japanese rice-ball’ shape [based on the Inorganic Crystal Structure Database (FIZ, Karlsruhe; http://www.fiz-informationsdienste.de/DB/icsd/www-recherche.htm) and the Cambridge Structural Database (CSD; Groom *et al.*, 2016 ▸), January 2022]

Formula	Building blocks of the ‘Japanese rice-ball’	Reference
Na_6_[Mo_120_O_366_(H_2_O)_48_H_12_{Pr(H_2_O)_5_}_6_]	12 {Mo_1_}, 6 {Mo_2_}, 12 {Mo_8_}, 6 {Pr}	Müller *et al.* (2000 ▸)
Na_6_[Mo_120_Ce_6_O_366_H_12_(H_2_O)_78_]	12 {Mo_1_}, 6 {Mo_2_}, 12 {Mo_8_}, 6 {Ce}	Duros *et al.* (2017 ▸)
[NH_4_]_4_[Mo_120_O_366_H_14_(H_2_O)_48_{La(H_2_O)_5_}_6_]	12 {Mo_1_}, 6 {Mo_2_}, 12 {Mo_8_}, 6 {La}	Yamase *et al.* (2006 ▸)
(C_5_H_14_N_2_O_2_)_2_[{Mo_8_O_26_}_0.5_ 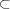 H_12_Mo_124_Ce_4_O_376_(H_2_O)_60_(C_5_H_13_N_2_O_2_)_6_]	12 {Mo_1_}, 8 {Mo_2_}, 12 {Mo_8_}, 4 {Ce}	Xuan *et al.* (2019 ▸)
Na_2_(C_10_H_17_N_5_O_4_)[Mo_122_Ce_5_O_371_(H_2_O)_69_H_12_(C_10_H_16_N_5_O_4_)_3_]	12 {Mo_1_}, 7 {Mo_2_}, 12 {Mo_8_}, 5 {Ce}	She *et al.* (2021 ▸)

**Table 2:** Experimental details

Crystal data	
Chemical formula	Na_4_[Mo_2_O_2_(OH)_4_(C_6_H_4_NO_2_)_2_]_2_[Mo_120_Ce_6_O_366_H_12_(OH)_2_(H_2_O)_76_]∼200H_2_O
*M*_r_	24391.76
Crystal system, space group	Monoclinic, *C*2/*c*
Temperature (K)	200
*a*, *b*, *c* (Å)	54.272 (10), 38.896 (7), 31.734 (6)
β (°)	112.145 (4)
*V* (Å^3^)	62047 (19)
*Z*	4
Radiation type	Mo *K*α
μ (mm^−1^)	2.97
Crystal size (mm)	0.18 × 0.15 × 0.08

Data collection	
Diffractometer	Bruker APEXII CCD
Absorption correction	Multi-scan (*SADABS*; Bruker, 2016 ▸)
*T*_min_, *T*_max_	0.583, 0.745
No. of measured, independent and observed [*I* > 2σ(*I*)] reflections	373860, 32484, 23243
*R*_int_	0.101
θ_max_ (°)	20.9
(sin θ/λ)_max_ (Å^−1^)	0.502

Refinement	
*R*[*F* ^2^ > 2σ(*F* ^2^)], *wR*(*F* ^2^), *S*	0.083, 0.234, 1.07
No. of reflections	32484
No. of parameters	2814
No. of restraints	239
H-atom treatment	H-atom parameters constrained
Δρ_max_, Δρ_min_ (e Å^−3^)	3.03, −2.19

Computer programs: *APEX2* (Bruker, 2016 ▸), *SAINT* (Bruker, 2016 ▸), *SHELXT2018* (Sheldrick, 2015*a* ▸), *PLATON* (Spek, 2020 ▸), *SHELXL2018* (Sheldrick, 2015*b* ▸), *OLEX2* (Dolomanov *et al.*, 2009 ▸), *DIAMOND* (Brandenburg, 2006 ▸) and *shelXle* (Hübschle *et al.*, 2011 ▸).

**Table 3:** Summary of the main vibrational bands observed for {Mo_2_(C_6_H_4_NO_2_)_2_}_2_{Mo_120_Ce_6_} Intensities are denoted as: *w* = weak, *m* = medium, *s* = strong, and *br* = broad.

Wavenumber (cm^−1^)	Assignment
3252 (*br*)	ν(O—H) of H_2_O
1606 (*m*)	δ(O—H) of H_2_O
1411 (*m*)	ν(C=N) 2-picolinic acid
1092 (*m*)	ν(C—N) 2-picolinic acid
967 (*m*)	ν(Mo=O) of **Ln-MB**
904 (*m*)	ν(Mo—O—Mo) of **Ln-MB**
866 (*m*)	ν(Mo—O—Mo) of **Ln-MB**
806 (*s*)	ν(Mo—O—Mo) of **Ln-MB**
746 (*s*)	ν(Mo—O—Mo) of **Ln-MB**
620 (*s*)	ν(Mo—O—Mo) of **Ln-MB**
528 (*s*)	ν(Mo—O—Mo) of **Ln-MB**
